# Magnitude and determinants of behavioral risk factors of non-communicable diseases among reproductive age women in Gofa and Basketo zones, Southern Ethiopia: a community-based cross-sectional study

**DOI:** 10.3389/fpubh.2025.1550043

**Published:** 2025-07-16

**Authors:** Markos Manote Domba, Terefe Gelibo Argefa, Bahiru Mulatu Kebede, Sewunet Sako

**Affiliations:** ^1^Public Health, Gofa Zone Health Department, South Ethiopia Regional State Health Bureau, Sawla, Ethiopia; ^2^Department of Public Health, Mailman School of Public Health, Columbia University, Addis Ababa, Ethiopia; ^3^Department of Public Health, Arba Minch College of Health Science, Arba Minch, Ethiopia; ^4^Department of Health Informatics, School of Public Health, College of Medicine and Health Sciences, Arba Minch University, Arba Minch, Ethiopia

**Keywords:** behavioral risk factors, non-communicable diseases, reproductive-aged women, Gofa, Basketo, South Ethiopia

## Abstract

**Background:**

As of 2019, around 2 billion reproductive-age women worldwide were impacted by NCDs and their associated risk factors. The purpose of this study is to investigate the magnitude and factors associated with behavioral risk factors among reproductive-age women in the Gofa and Basketo zones of Southern Ethiopia, with the goal of improving care options for women with NCDs before, during, and after pregnancy.

**Methods:**

A community-based survey was conducted using instruments adapted and developed from the WHO STEPS surveillance manual and various literatures on chronic disease risk factors. A multistage cluster sampling approach was employed to select individuals from the two zones. Statistical analysis was performed using the Statistical Package for the Social Sciences (SPSS) software. Descriptive statistics, bivariate analysis, and multivariate logistic regression were conducted. Associations with a *p* ≤ 0.05 were considered statistically significant.

**Results:**

Approximately 90.9% (95% CI: 89.9–90.2) of participants exhibited one or more behavioral risk factors. High dietary salt intake was the most common risk factor accounting for 90.0%. Statistically significant associations with the co-occurrence of behavioral risk factors were found among women in younger age groups, rural residents, Gofa zone residents, widowed/divorced or single individuals, merchants, housewives, individuals from households with a lower wealth index, those with lower educational attainment, and having a family history of NCDs. Conversely, being a government employee, mass media user, having good knowledge of NCD risk factors, having social support, and being a member of the functional Women Development Army (WDA) were identified as protective factors.

**Conclusion:**

The escalation of behavioral risk factors is concerning, highlighting the urgent need for targeted community-based interventions. It is recommended to prioritize younger age groups, rural residents, those with lower wealth status, and lower educational attainment. Implementing family-oriented changes and strengthening healthcare systems are crucial. Additionally, addressing policy and socio-political factors influencing the rise of NCD risk factors is essential.

## Introduction

Non-communicable diseases (NCDs) are non-transmissible diseases of often long duration (1). NCDs are the dominant cause of global morbidity and mortality, especially in developing countries (2, 3). The rising burden of NCDs is coupled with unmet needs of sexual and reproductive health services in sub-Saharan Africa (SSA) (4). They are doubled among reproductive-age women in many African countries (5, 6), and are expected to exceed infectious diseases as major causes of morbidity and mortality in Africa by the year 2035 (7).

NCDs are mostly linked with behavioral (such as tobacco use, harmful use of alcohol, high dietary salt intake, and chewing khat) and metabolic risk factors (8). Maternal health and NCDs are linked in a vicious cycle as poor maternal health raises the risk of NCDs in kids and succeeding generations, whereas NCDs cause poor mother health (9–13). Furthermore, women with NCD risk factors hurt their reproductive health as well as fetal health (14). It also impedes progress toward sustainable development goals (SDG), mainly SDGs 1, 3, and 5 (15). Hence, it requires special attention to effectively address NCDs and their key risk factors since NCDs and their risk factors are inseparably related to maternal and child health (9, 16).

Globally, two-thirds (19 million) of all women’s deaths were caused by NCDs. Similarly, the study found that 2 billion women of reproductive age were impacted by NCDs and their risk factors. Furthermore, many younger women are already at danger because NCDs cause a high number of deaths among the older age (over 50) (17, 18).

According to one study, out of the 130 million pregnancies that result in live births worldwide each year, 21 million are affected by hyperglycemia, 7–8 million by hypertension, 42 million by maternal overweight and obesity, 26 million by maternal malnutrition, and 56 million by maternal anemia. These disorders increase the risk of NCD (diabetes, obesity, hypertension, cardiovascular disease, and stroke) for both the mother and the newborn, in addition to unfavorable pregnancy outcomes (12).

In Ethiopia, evidence shows that 95% of the study population was found with 1–2 NCD risk factors (19) and 98.4% have at least one risk factor. This indicates that the burden of NCDs is likely to become unbearable in the future in Ethiopia. According to the WHO 2022 report, Ethiopia had 271,300 (43%) NCD deaths despite implementing various interventions such as increased excise taxes and prices, smoke-free policies, bans on advertising, promotion, and sponsorship of tobacco and harmful alcohol use, public education and awareness campaigns on physical activity, and so on (20).

Tackling NCDs in women needs a systematic understanding of major NCD risk factors and determinants (21, 22). Despite the increasing burden of the disease in Ethiopia, there is a paucity of recent data on behavioral risk factors and determinants focusing on reproductive-age women, especially in the peripheral setup of the country like Gofa and Basketo. Therefore, this study aimed to address the above gaps and, to advocate the policymakers and regional government to prevent NCD risk factors among women of reproductive age.

## Methods

### Study setting and period

The study was conducted in Gofa and Basketo zones, Southern Ethiopia from September 6, 2022 to December 9, 2022.

### Study design

A community-based cross-sectional study was conducted by the WHO a stepwise approach to the surveillance of NCD risk factors.

### Study population

All women of reproductive age residing in the Gofa and Basketo zones during the data collection period were eligible if they considered the study area their permanent residence for at least 6 months. Exclusions were made for non-permanent residents, pregnant women, individuals institutionalized in hospitals, prisons, nursing homes, or similar facilities, as well as those residing primarily in military camps or dormitories. Additionally, individuals who were critically ill, mentally disabled, or physically disabled and unsuitable for physical participation were excluded.

### Sample size determination and sampling technique

A mix of sampling approach: stratified, multi-stage cluster sampling, systematic random sampling and Kish method were employed to select the study population and the study participants. Gofa and Basketo zones have 13 districts and each district stratified into rural (193 kebeles) and urban (62 kebeles) yielding 255 sampling strata. Kebeles are the lowest administrative structures in Ethiopia. Kish method was employed to select only one eligible participant in the selected HH. The method was used to randomize whom to interview within a household when going door to door and eligible participants in each household were ranked in order of decreasing age (23). The WHO regional office tools for assessing operational district health systems in Africa recommend that for districts numbering between 10 and 19, 50% of them could be sampled (24). The sample size was determined using a single proportion formula with the following parameters: Z-score = 1.96, Proportion = 50%, Margin of error = 0.05, Design effect = 3.35 and non-response rate = 10%, resulting in a total sample size of 1,416 respondents.

### Study variables and measurement

#### Dependent variable

Behavioral risk factors of non-communicable disease including current tobacco use, alcohol consumption, high dietary salt intake, and chewing khat. Current tobacco use, alcohol consumption, high dietary salt intake, and chewing khat were measured with questions: Do you currently smoke any tobacco products, such as cigarettes, cigars or pipes and/or do you currently use smokeless tobacco such as snuff, chewing tobacco? Have you consumed any alcoholic beverages within the past 30 days (25). High dietary salt was measured using two questions. Do you eat freshly prepared food and/or processed foods having high salt content? And/or do you always add salt or salty sauce to your food (before eating or while they are eating)? khat chewing was also measured using two questions. The first measurement was employed by asking the women whether they had ever chewed khat in their lifetime, and to determine current chewing, whether they had chewed khat in the past 30 days prior to the survey (26).

#### Independent variable

Socio-demographic and cultural variables include age, place of residence, family history, family size, educational status, marital status, occupation of women, social support to NCD prevention and wealth status of household. Knowledge-related factors/variables include Knowledge of NCD risk factors, getting advice from health professionals, and using mass media. Structural factors include membership in the functional women’s development army.

In this study, social support for NCD prevention include material aid, emotional and/or informational support provided to reproductive age women by nonprofessionals in the context of both formal support groups and informal helping relationships.

Wealth status was derived from the wealth index (five quintiles in the data set; poorest, poor, middle, rich and the richest) for the households. The variables included to calculate the index were main material of the walls, roofing, floor, separate room for cooking, type of fuel household mainly use for cooking, kind of toilet facility household use, household’s ownership of phone, radio, Television, mattress, bed, watch, stove, table, chair, beehive, ox, caw, hen, motorcycle and Generator.

### Data collection tool

The data collection instrument is a questionnaire developed with the adaptation of the WHO Stepwise Surveillance questionnaire. This questionnaire was translated into *Gofatho* and Basket languages and subsequently back-translated into English to ensure accuracy. Socio-demographic, knowledge, and health system-related data were collected.

### Data collection technique

All selected data collectors received 2 days of training on data collection before the survey. The training included instruction on using the instrument, interactive discussions, and conducting physical measurements. The interviewer-administered questionnaire covers socio-demographic information, knowledge, health system-related questions, and NCD risk factors. The assessment of variables was made according to suggestions by different scholars or standard guidelines such as the measurement of socio-demographic variables (5), wealth index (27, 28), variables for evaluating behavioral risk factors (tobacco use and alcohol consumption) (25).

### Data quality management

To ensure data quality, the questionnaire underwent necessary translation and pretesting. After data collection, thorough checks for completeness and consistency were conducted, and coding was performed by both the supervisor and principal investigator.

### Weighting of data

Data was weighted because it comprises sample of target population. The sample weighting was carried out to correct differences in distribution of the sample age and area of residence versus the target population and probabilities of selection. The sample weight for each case in the sample accounts for the number of cases in the sampling frame. The product of the sample weight was used in weighted analysis.

### Data analysis

SPSS software version 25 was used for data analysis. Descriptive weighted analysis was conducted along with complex sample analysis. The presence of associations was assessed using bivariate analysis, followed by multivariate logistic regression analysis to identify independent predictors. The strength of the association was estimated using odds ratios and their 95% confidence intervals. Associations with a *p* ≤ 0.05 were considered statistically significant.

### Ethical considerations

The survey protocol received ethical clearance from the institutional review boards of the Southern Regional State Public Health Institute and Selinus University of Science and Literature. Written informed consent was obtained from all eligible participants using a paper-based consent form. Participants indicated their consent by signing or making a mark on the printed consent form, which was retained by both the participant and the data collector.

For household participation, a designated head of household provided written consent, after which individual members were rostered for a household interview. For minors aged 15–17, parental or guardian permission was obtained, followed by assent from the participant.

## Results

### Socio-demographic characteristics of participants

The participants had an average age of 28.9 years with a standard deviation of 7.5 years, and 41.5% were aged between 25 and 34 years. A significant portion (48.2%) had no formal education, while the majorities (93.4%) were married. Most participants were from the Gofa zone (93.3%) and rural areas (83.3%). Regarding wealth distribution, approximately 20.5, 21.1, and 20.6% of participants were categorized into the poorest, poorer, and middle wealth quintiles, respectively. Housewives comprised the largest occupational group, accounting for 72% of participants. For further socio-demographic details, please refer to Table 1.

**Table 1:** Socio-demographic characteristics of participants.

Characteristics	Un-weighted count	Weighted percent
Age		
15–24	400	29.4
25–34	599	41.5
≥35	405	29.1
Educational status		
Illiterate	519	38.6
No formal education but able to read and write	147	9.6
Primary education	400	28.7
Secondary education and above	338	23.1
Marital status		
Married	1,297	93.4
Single	68	4.4
Widowed/Divorced	39	2.1
Residence		
Urban	393	16.2
Rural	1,011	83.8
Zone		
Gofa	1,244	93.3
Basketo	160	6.7
Wealth index		
Poorest	288	20.5
Poorer	286	21.1
Middle	277	20.6
Richer	280	19.5
Richest	273	18.3
Occupational status		
Housewife	981	71.9
Merchant	172	12.7
Government employee	76	4.4
Other*	175	11.0
Family size		
≤ 4	544	38.4
>4	860	61.6
Family history of NCDs		
No	1,292	92.7
Yes	112	7.3
Social support to prevent NCD risk factors		
No	846	60.8
Yes	558	39.2

*Others in occupational status: daily laborers, students, and maidservants.

Regarding knowledge of NCD risk factors, only 34.6% of the study participants had good knowledge; but the vast majority had poor knowledge.

### Prevalence of behavioral risk factors

The prevalence of behavioral non-communicable disease risk factors (tobacco use, alcohol consumption, high salt intake, and chewing khat) among different socio-demographic sub-groups is presented in Table 2.

**Table 2:** Prevalence of modifiable behavioral non-communicable disease risk factors by background characteristics of reproductive-aged women.

Socio-demographic characteristics	Current tobacco use *n* (%)	Alcohol use *n* (%)	High salt intake *n* (%)	Chewing khat *n* (%)
Age				
15–24	11 (2.8)	32 (8.0)	363 (90.7)	12 (3.0)
25–34	6 (1.0)	46 (7.7)	522 (87.1)	28 (4.7)
≥35	7 (1.7)	29 (7.2)	347 (85.7)	32 (8.0)
*P*-value	0.113	0.902	0.075	0.001^*^
Educational status				
Illiterate	13 (2.5)	43 (8.3)	463 (89.2)	28 (5.4)
Read and write	2 (1.4)	9 (6.1)	128 (87.1)	0
Primary education	9 (2.3)	32 (8.0)	349 (87.3)	31 (7.7)
Secondary education and above	0	23 (6.8)	292 (86.4)	13 (3.8)
*P*-value	0.035	0.754	0.622	0.001^*^
Marital status				
Married	24 (1.9)	98 (7.6)	1,137 (87.7)	72 (5.5)
Single	0	6 (8.8)	61 (89.7)	0
Widowed/Divorced	0	3 (7.7)	34 (87.2)	0
*P*-value	0.365	0.929	0.877	0.999
Residence				
Urban	4 (1.0)	18 (4.6)	318 (80.9)	37 (9.4)
Rural	20 (2.0)	89 (8.8)	914 (90.4)	35 (3.5)
*P*-value	0.213	0.007^*^	<0.001*	0.001^*^
Zone				
Gofa	24 (1.9)	77 (6.2)	1,092 (87.8)	62 (5.1)
Basketo	0	30 (18.8)	140 (87.5)	10 (6.2)
*P*-value	0.076	<0.001^*^	0.919	0.001^*^
Wealth index				
Poorest	4 (1.4)	15 (5.2)	256 (88.9)	8 (2.8)
Poorer	3 (1.0)	14 (4.9)	246 (86.0)	8 (2.8)
Middle	0	23 (8.3)	240 (86.6)	20 (7.2)
Richer	11 (3.9)	28 (10.0)	257 (91.8)	20 (7.1)
Richest	6 (2.2)	27 (9.9)	233 (85.3)	16 (5.9)
*P*-value	0.006^*^	0.043^*^	0.127	<0.001*
Occupational status				
Housewife	20 (2.0)	69 (7.0)	854 (87.1)	64 (6.5)
Merchant	3 (1.7)	21 (12.2)	162 (94.2)	0
Government employee	1 (1.3)	5 (6.6)	62 (81.6)	0
Other	0	12 (6.9)	154 (88.0)	8 (4.6)
*P*-value	0.290	0.117	0.021^*^	0.102
Family size				
≤4	6 (1.1)	34 (6.3)	476 (87.5)	32 (5.9)
>4	18 (2.1)	73 (8.5)	756 (87.9)	40 (4.6)
*P*-value	0.163	0.124	0.821	0.001*

*P <0.05, **P <0.001, ***P <0.0001.

#### Tobacco use

The prevalence of current tobacco use was 2.0% (95% CI: 1.9–2.0). Among women aged 15–24, the prevalence was higher at 2.8% (95% CI: 1.5–4.7) compared to women aged 35 and above, where it was 1.7% (95% CI: 0.8–3.4). Tobacco use was also more prevalent among rural residents (2.0, 95% CI: 1.3–3.0) than urban residents (1.0, 95% CI: 0.3–2.4). Furthermore, the prevalence of tobacco use was higher among women with more than four family members (2.1, 95% CI: 1.3–3.2) compared to those with four or fewer (1.1, 95% CI: 0.5–2.3). However, it was significantly lower (*p* = 0.006) among the poorest wealth quintile (1.4, 95% CI: 0.5–3.3) compared to the richest (2.2, 95% CI: 0.9–4.5).

#### Alcohol consumption

The overall prevalence of consuming alcoholic drinks within 30 days was 7.0% (95% CI 6.9–7.2). The prevalence of alcohol consumption was significantly higher (*p* = 0.007) among rural residents (8.8, 95% CI: 7.2–10.7) compared to urban women (4.6, 95% CI: 2.8–7.0). It was also significantly higher (*p* < 0.001) among Basketo zone residents (18.8, 95% CI: 13.3–25.3) compared to Gofa zone residents (6.2, 95% CI: 5.0–7.6). The prevalence of alcoholic drinks within 30 days was lower among the poorest (5.2, 95% CI: 3.1–8.2) or poorer (4.9, 95% CI: 2.8–7.9) compared to the richest (9.9, 95% CI: 6.8–13.9). Additionally, the prevalence of alcohol consumption was higher among women from households with family sizes >4 (8.5, 95% CI: 6.8–10.5) compared to women from households with family sizes of 4 or fewer (6.3, 95% CI: 4.4–8.5).

#### High salt intake

The prevalence of high dietary salt intake was the most common non-communicable disease risk factor among the study participants, at 90.0% (95% CI: 89.9–90.2). High dietary salt intake was inversely related to the age of women; the prevalence among women aged 15–24, 25–34, and > = 35 years were 90.8% (95% CI: 87.6–93.3), 87.1% (95% CI: 84.3–89.6), and 85.7% (95% CI: 82.0–88.8), respectively. The prevalence of high dietary salt intake was lower among urban residents (80.9, 95% CI: 76.8–84.6) compared to rural residents (90.4, 95% CI: 88.5–92.1).

#### Chewing khat

The overall prevalence of chewing khat was 4.4% (95% CI 1.8–10.5). The prevalence was significantly higher (*p* < 0.004) among urban residents (9.4, 95% CI: 5.0–15.3) compared to rural residents. It was also higher among women residing in the Basketo zone (6.2%) compared to those in the Gofa zone (5.1, 95% CI: 1.6–11.0).

### Prevalence of combined behavioral risk factors

**Figure 1:**
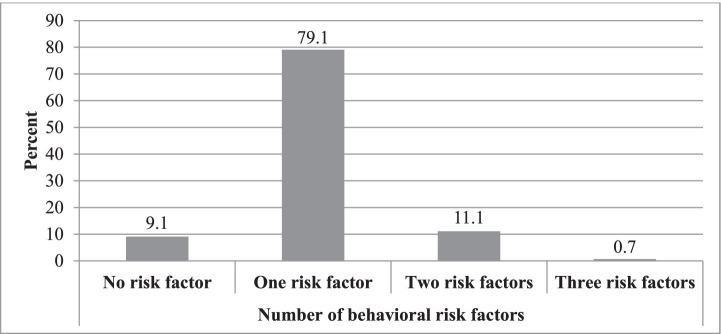
Prevalence by the number of behavioral risk factors among reproductive age group women in Gofa and Basketo zones, Southern Ethiopia.

About 90.9% of women in the reproductive age group had at least one risk factor (see Figure 1).

### Factors associated with non-communicable diseases risk factors

Binary logistic regression assessed associations between outcomes and independent variables (Tables 3–7). Significant associations were found between current tobacco use and age, residence, wealth index, family size, mass media use, health professional advice, and membership in a functional WDA in bivariate analysis. Age, education, residence, wealth index, occupation, family size, and mass media use were associated with drinking alcohol within 30 days. High dietary salt intake was associated with age, education, residence, zone, wealth index, occupation, mass media use, and family history of NCDs, social support, WDA membership, and knowledge of risk factors. Chewing khat was associated with age, education, residence, zone, wealth status, family size, mass media use, WDA membership, health professional advice, social support, and family history of NCDs. Variables with *p*-values < 0.25 in binary logistic regression were included in multivariable models to control for confounding. Tables 3–7 present factors associated with these behavioral risk factors. For example, women aged 25–34 years (AOR: 0.21, 95% CI 0.19–0.23) and ≥ 35 years (AOR: 0.21, 95% CI 0.18–0.23) were five times less likely than those aged 15–24 years to use tobacco. Non-users of mass media were about twice as likely to use tobacco (AOR: 1.82, 95% CI 1.66–2.01) compared to mass media users.

**Table 3:** Bivariate and multivariate logistic regression of tobacco use and independent variables among reproductive-age women.

Candidate variables	COR (95% CI)	AOR (95% CI)
Age		
15–24	1	1
25–34	0.34 (0.31, 0.37)**	0.21 (0.19, 0.23)**
≥35	0.35 (0.32, 0.39)**	0.21 (0.18, 0.23)**
Residence		
Urban	1	1
Rural	3.03 (2.61, 3.54)**	2.79 (2.37, 3.28)**
Wealth index		
Poorest	0.58 (0.53, 0.65)**	0.48 (0.43, 0.53)**
Poorer	0.46 (0.41, 0.52)**	0.41 (0.35, 0.45)**
Middle	0.001 (0.01, 2.50)**	0.00 (0.00, 3.35)
Richer	1.18 (1.07, 1.27)**	0.94 (0.85, 1.04)
Richest	1	1
Family size		
≤4	1	1
>4	1.54 (1.42, 1.68)	2.79 (2.53, 3.06)**
Do you use mass media		
No	1.57 (1.44,1.72)**	1.82 (1.66, 2.01)**
Yes	1	1
Getting advice from health professionals		
No	1	1
Yes	1.22 (1.12, 1.33)**	0.76 (0.69, 0.83)**

*P < 0.05, **P < 0.001, ***P < 0.0001. COR, Crude Odds Ratio; CI, confidence interval; AOR, Adjusted Odds Ratio.

**Table 4:** Bivariate and multivariate logistic regression of alcohol use and independent variables among reproductive-age women.

Candidate variables	COR (95% CI)	AOR (95% CI)
Age		
15–24	1.17 (1.11, 1.24)*	1.85 (1.73, 1.97)*
25–34	1.24 (1.18, 1.31)*	1.32 (1.25, 1.39)*
≥35	1	1
	1	1
Educational status		
Illiterate	1	1
No formal education but able to read and write	0.63 (0.58, 0.69)*	0.54 (0.51, 0.59)*
Primary education	0.91 (0.85, 0.94)*	1.01 (0.95, 1.06)
Secondary education and above	0.83 (0.79, 0.88)*	0.82 (0.76, 0.87)*
Residence		
Urban	0.60 (0.566, 0.64)*	0.96 (0.89, 1.02)
Rural	1	1
Zone		
Gofa	1	1
Basketo	3.48 (3.29, 3.68)*	4.01 (3.77, 4.26)*
Wealth index		
Poorest	1	1
Poorer	0.98 (0.90, 1.07)	0.92 (0.85, 1.01)
Middle	2.25 (2.11, 2.42)*	2.31 (2.15, 2.49)*
Richer	2.71 (2.51, 2.91)*	3.20 (2.97, 3.45)*
Richest	2.65 (2.47, 2.85)*	3.10 (2.88, 3.35)
Occupational status		
Housewife	1.33 (1.23, 1.43)*	1.67 (1.53, 1.82)*
Merchant	2.75 (2.52, 2.99)*	3.91 (3.56, 4.29)*
Government employee	1.92 (1.72, 2.15)*	1.85 (1.64, 2.09)*
Other	1	1
Family size		
<= 4	1	1
>4	1.22 (1.17, 1.28)*	1.12 (1.07, 1.18)*
Do you use mass media?		
No	1.41 (1.34, 1.47)*	1.83 (1.74, 1.94)*
Yes	1	1
Family history of NCDs		
No	1	1
Yes	1.53 (1.43, 1.63)*	1.42 (1.32, 1.54)*
Is your WDA functional?		
No	1.05 (1.01, 1.10)*	1.23 (1.18, 1.29)
Yes	1	1

^*^Significant at *p* < 0.05.

**Table 5:** Bivariate and multivariate logistic regression of high dietary salt intake and independent variables among reproductive-age women.

Candidate variables	COR (95% CI)	AOR (95% CI)
Age		
15–24	1.66 (1.58, 1.74)*	1.65 (1.57,1.74)*
25–34	1.09 (1.05, 1.14)*	1.11 (1.07, 1.16)*
> = 35	1	1
Educational status		
Unable to read and write	1.12 (1.07, 1.17)*	1.21 (1.15, 1.28)*
No formal education but able to read and write	0.98 (0.92, 1.05)	1.05 (0.98, 1.12)
Primary education	1.09 (1.05, 1.15)*	1.14 (1.08, 1.20)*
Secondary education and above	1	1
Marital status		
Married	0.97 (0.86, 1.09)	0.74 (0.65, 0.85)*
Single	1.12 (0.97, 1.30)	0.77 (0.66, 0.91)*
Widowed/Divorced	1	1
Residence		
Urban	1	1
Rural	3.18 (3.06, 3.30)*	2.98 (2.86, 3.11)*
Zone		
Gofa	1.31 (1.23, 1.40)*	1.83 (1.71, 1.96)*
Basketo	1	1
Wealth index		
Poorest	1.41 (1.34, 1.49)*	1.25 (1.18, 1.33)*
Poorer	1.06 (1.01, 1.12)*	0.94 (0.89, 0.99)*
Middle	1.20 (1.13, 1.26)*	1.06 (1.00, 1.12)*
Richer	1.78 (1.68, 1.89)*	1.65 (1.55, 1.75)*
Richest	1	1
Occupational status		
Housewife	1.05 (0.99, 1.11)	0.96 (0.91, 1.02)
Merchant	2.76 (2.53, 3.01)*	2.51 (2.29, 2.75)*
Government employee	0.77 (0.70, 0.84)*	0.81 (0.73, 0.89)*
Other	1	1
Do you use mass media		
No	1.35 (1.30, 1.40)*	1.17 (1.12, 1.22)*
Yes	1	1
Family history of NCDs		
No	1	1
Yes	1.28 (1.19, 1.38)*	1.63 (1.50, 1.76)*
Social support to prevent NCD risk factors		
No	0.89 (0.86, 0.93)*	1.33 (1.28, 1.38)
Yes	1	1
Is your WDA functional?		
No	1	1
Yes	1.11 (1.07,1.15)*	1.03 (0.99, 1.08)
Knowledge on risk factors		
<60% (i.e., 4.2)	1.35 (1.30, 1.40)	1.03 (0.99, 1.07)
≥60% (i.e., 4.2)	1	1

*Significant at *p* < 0.05.

**Table 6:** Bivariate and multivariate logistic regression of chewing khat and independent variables among reproductive age women.

Candidate variables	COR (95% CI)	AOR (95% CI)
Age		
15–24	0.43 (0.41, 0.46)*	0.63 (0.58, 0.69)*
25–34	0.38 (0.36, 0.41)*	0.36 (0.34, 0.38)*
> = 35	1	1
Educational status		
Illiterate	1.34 (1.25, 1.43)*	2.56 (2.36, 2.78)*
No formal education but able to read and write	n/a	n/a
Primary education	1.60 (1.49, 1.72)*	3.72 (3.43, 4.04)*
Secondary education and above	1	1
Residence		
Urban	2.71 (2.55, 2.85)*	3.25 (3.03, 3.48)*
Rural	1	1
Zone		
Gofa	1	1
Basketo	1.50 (1.37, 1.64)*	1.78 (1.62, 1.96)*
Wealth index		
Poorest	1	1
Poorer	1.55 (1.36, 1.76)*	2.23 (1.95, 2.55)*
Middle	3.99 (3.56, 4.48)*	5.47 (4.85, 6.17)*
Richer	7.00 (6.27, 7.82)*	14.04 (12.44, 15.84)*
Richest	4.86 (4.33, 5.44)*	6.62 (5.85, 7.51)*
Family size		
<= 4	1.26 (1.19, 1.32)*	2.39 (2.24, 2.55)*
>4	1	1
Do you use mass media		
No	0.563 (0.53, 0.59)*	1.26 (1.18, 1.35)*
Yes	1	1
Family history of NCDs		
No	1	1
Yes	4.11 (3.86, 4.38)*	4.76 (4.41, 5.14)*
Getting advice from health professionals		
No	1.99 (1.89, 2.09)*	1.34 (1.25, 1.43)*
Yes	1	1
Social support to prevent NCD risk factors		
No	1.82 (1.73, 1.91)*	1.23 (1.16, 1.31)*
Yes	1	1
Is your WDA functional?		
No	1.25 (1.19, 1.32)*	1.35 (1.27, 1.44)*
Yes	1	1

*Significant at *P* < 0.05.

**Table 7:** Bivariate and multivariate logistic regression of clustering of behavioral risk factors and independent variables among reproductive-age women.

Variables	COR (95% CI)	AOR (95% CI)
Age		
15–24	1.62 (1.55, 1.71)*	1.63 (1.55, 1.73)*
25–34	1.11 (1.06, 1.15)*	1.13 (1.09, 1.18)*
≥35	1	1
Educational status		
Illiterate	1.11 (1.06, 1.16)*	1.12 (1.06, 1.19)*
No formal education but able to read and write	0.89 (0.84, 0.95)*	0.91 (0.84, 0.97)*
Primary education	1.19 (1.13, 1.25)*	1.21 (1.15, 1.28)*
Secondary education and above	1	1
Marital status		
Married	1	1
Single	1.19 (1.09, 1.31)*	1.18 (1.06, 1.31)*
Widowed/Divorced	1.47 (1.27, 1.70)*	1.98 (1.70, 2.30)*
Zone		
Gofa	1.20 (1.12, 1.29)*	1.65 (1.54, 1.77)*
Basketo	1	1
Residence		
Urban	1	1
Rural	2.92 (2.81, 3.04)*	2.69 (2.57, 2.81)*
Wealth index		
Poorest	1.36 (1.29, 1.45)*	1.18 (1.11, 1.26)*
Poorer	0.94 (0.89, 0.99)*	0.80 (0.76, 0.85)*
Middle	1.17 (1.10, 1.24)*	0.99 (0.94, 1.05)
Richer	1.70 (1.60, 1.82)*	1.59 (1.51, 1.70)*
Richest	1	1
Occupational status		
Housewife	1.13 (1.07, 1.19)*	1.14 (1.07, 1.21)*
Merchant	2.64 (2.42, 2.88)*	2.61 (2.36, 2.85)*
Government employee	0.84 (0.77, 0.92)*	0.85 (0.76, 0.94)*
Other	1	1
Do you use mass media		
No	1.37 (1.32, 1.42)*	1.30 (1.25, 1.36)*
Yes	1	1
Social support for prevention of NCD risk factors		
No	1.24 (1.21, 1.29)*	1.47 (1.41, 1.53)*
Yes	1	1
Family history of NCDs		
No	1	1
Yes	1.57 (1.45, 1.71)*	2.05 (1.88, 2.24)*
Is your WDA functional?		
No	1.05 (1.01, 1.09)*	10.98 (0.94, 1.03)
Yes	1	1
Knowledge on risk factors		
<60% (i.e., 4.2)	1.27 (1.22, 1.32)*	1.10 (1.06, 1.15)*
≥60% (i.e., 4.2)	1	1

*Significant at *p* < 0.05.

Current tobacco use was approximately three times more likely among rural residents (AOR: 2.79, 95% CI 2.37–3.28) compared to urban residents. Women from the poorest (AOR: 0.48, 95% CI 0.43–0.53) and poorer (AOR: 0.48, 95% CI 0.43–0.53) households were about half as likely to use tobacco compared to those from the richest households. Additionally, current tobacco use was about three times more likely among women from households with family size >4 (AOR: 2.79, 95% CI 2.53–3.06) compared to those with family size ≤4. Women who received advice from health professionals were less likely to use tobacco (AOR: 0.76, 95% CI 0.69–0.83) than those who did not receive such advice.

Alcohol use within the last 30 days was more likely among women aged 15–24 (AOR: 1.86, 95% CI 1.74–1.98) and 25–34 (AOR: 1.32, 95% CI 1.25–1.39) compared to women ≥35 years old. Literate women (AOR: 0.54, 95% CI 0.49–0.59) or who had secondary education (AOR: 0.81, 95% CI 0.76–0.87) were less likely to use alcohol than illiterate women. Residents of the Basketo area were four times more likely to drink alcohol (AOR: 4.04, 95% CI 3.80–4.29) than Gofa residents. Participants who did not use mass media were about two times more likely to use alcohol (AOR: 1.85, 95% CI 1.75–1.95) compared to mass media users.

Women in the middle quartile (AOR: 2.32, 95% CI 2.15–2.51), richer (AOR: 3.21, 95% CI 2.98–3.46), and richest (AOR: 3.10, 95% CI 2.88–3.34) households were more likely to drink alcohol compared to the poorest. However, women from poorer households were slightly less likely to drink alcohol (AOR: 0.92, 95% CI 0.85–1.01) compared to the poorest. Housewives (AOR: 1.68, 95% CI 1.54–1.83), merchants (AOR: 3.93, 95% CI 3.58–4.31), and government employees (AOR: 1.85, 95% CI 1.64–2.09) were more likely to drink alcohol compared to other occupations such as daily laborers, maidservants, and students.

Women from households with family size >4 (AOR: 1.12, 95% CI 1.07–1.18) were more likely to drink alcohol compared to those from households with ≤4 family members. Women with a family history of NCDs were more likely to use alcohol (AOR: 1.42, 95% CI 1.32–1.54) compared to those without a family history. Women who were not members of a WDA were more likely to drink alcohol compared to those who were members (AOR: 1.23, 95% CI 1.18–1.29).

The odds of having high dietary salt intake were higher among women aged 15–24 years (AOR: 1.65, 95% CI 1.57–1.74) and 25–34 years (AOR: 1.11, 95% CI 1.07–1.16) compared to those aged ≥35 years. Women who were unable to read and write (AOR: 1.21, 95% CI 1.15–1.28) or had primary education (AOR: 1.14, 95% CI 1.08–1.20) were more likely to use high dietary salt compared to those with secondary education.

Rural residents were about three times more likely to use high dietary salt (AOR: 2.98, 95% CI 2.86–3.11) than urban residents. Residents of the Gofa area were about two times more likely to use high dietary salt (AOR: 1.83, 95% CI 1.71–1.96) compared to those in the Basketo zone. Women from the poorest (AOR: 1.25, 95% CI 1.18–1.33), middle (AOR: 1.06, 95% CI 1.00–1.12), and richer (AOR: 1.65, 95% CI 1.55–1.75) households were more likely to use high dietary salt compared to the richest, while it was less likely among those from poorer households (AOR: 0.94, 95% CI 0.89–0.99) compared to the richest.

Merchants were about two times more likely to use high dietary salt (AOR: 2.51, 95% CI 2.29–2.75) compared to other occupations (maidservants, daily laborers, and students), but it was less likely among government employees (AOR: 0.81, 95% CI 0.73–0.89) compared to others. Non-mass media users were more likely to use high dietary salt (AOR: 1.17, 95% CI 1.12–1.22) compared to mass media users.

Women with a family history of NCDs were more likely to use high dietary salt (AOR: 1.63, 95% CI 1.50–1.76) compared to those without a family history. Women without social support were more likely to use high dietary salt (AOR: 1.33, 95% CI 1.28–1.38) compared to those with social support.

The odds of chewing khat were less likely among women aged 15–24 years (AOR: 0.63, 95% CI 0.58–0.69) and 25–34 years (AOR: 0.36, 95% CI 0.34–0.38) compared to those aged ≥35 years. Illiterate women (AOR: 2.56, 95% CI 2.36–2.78) and those with primary education (AOR: 3.72, 95% CI 3.43–4.04) were more likely to chew khat compared to those with secondary education.

Urban residents were about three times more likely to chew khat (AOR: 3.25, 95% CI 3.03–3.48) compared to rural residents, and residents of the Basketo area were about two times more likely (AOR: 1.78, 95% CI 1.62–1.96) compared to those in the Gofa zone. Women from poorer (AOR: 2.23, 95% CI 1.95–2.55), middle (AOR: 5.47, 95% CI 4.85–6.17), richer (AOR: 14.04, 95% CI 12.44–15.84), and richest (AOR: 6.62, 95% CI 5.85–7.51) households were more likely to chew khat compared to the poorest. However, women from households with smaller family sizes (≤4) were more likely to chew khat (AOR: 2.39, 95% CI 2.24–2.55) compared to those with larger family sizes (>4).

Non-mass media users were more likely to chew khat (AOR: 1.26, 95% CI 1.18–1.35) compared to mass media users. Chewing khat was about five times more likely among women with a family history of NCDs (AOR: 4.76, 95% CI 4.41–5.14) compared to those without a family history. Women who did not receive health professional advice (AOR: 1.34, 95% CI 1.25–1.43), were not members of a functional WDA (AOR: 1.35, 95% CI 1.27–1.44), or lacked social support (AOR: 1.23, 95% CI 1.16–1.31) were more likely to chew khat compared to their counterparts.

### Clustering of behavioral NCD risk factors

Over 90 % of participants had at least one behavioral risk factor, with about 12 % having two or more. The prevalence of behavioral risk factors among reproductive-age women is shown in Figure 1 and Table 7 presents crude and adjusted odds ratios for risk factor clustering in the Gofa and Basketo zones.

Logistic regression analysis revealed that women aged 15–24 (AOR: 1.63, 95% CI 1.55–1.73) and 25–34 (AOR: 1.13, 95% CI 1.09–1.18) were more likely to experience co-occurrence of risk factors compared to those aged ≥35 years. Illiterate women (AOR: 1.12, 95% CI 1.06–1.19) and those with primary school education (AOR: 1.21, 95% CI 1.15–1.28) were more likely to have risk factor clustering compared to those with secondary education. However, women who could read and write (AOR: 0.91, 95% CI 0.84–0.97) were less likely to experience risk factor clustering compared to those with secondary education.

Widowed/divorced (AOR: 1.98, 95% CI 1.70–2.30) and single women (AOR: 1.18, 95% CI 1.06–1.31) were more likely to have risk factor clustering compared to married women. Additionally, women residing in the Gofa (AOR: 1.65, 95% CI 1.54–1.77) and rural areas (AOR: 2.69, 95% CI 2.57–2.81) were more likely to experience risk factor clustering.

Wealth status also showed associations, with participants in the poorest (AOR: 1.18, 95% CI 1.11–1.26) and richer (AOR: 1.59, 95% CI 1.51–1.70) quartiles being more likely to have risk factor clustering compared to the richest quartile, while those in the poorer quartile (AOR: 0.80, 95% CI 0.76–0.85) were less likely. Housewives (AOR: 1.14, 95% CI 1.07–1.21) and merchants (AOR: 2.61, 95% CI 2.36–2.85) were more likely to have risk factor clustering, whereas government employees (AOR: 0.85, 95% CI 0.76–0.94) were less likely.

Not being a member of a functional WDA (AOR: 1.98, 95% CI 1.94–2.03), lack of social support (AOR: 1.47, 95% CI 1.41–1.53), having family members with NCDs (AOR: 2.05, 95% CI 1.88–2.24), not using mass media (AOR: 1.30, 95% CI 1.25–1.36), and poor knowledge of NCD risk factors (AOR: 1.10, 95% CI 1.06–1.15) were associated with a higher probability of risk factor clustering.

## Discussion

The study generated data on the prevalence of behavioral NCD risk factors, including current tobacco use, alcohol consumption within 30 days, high dietary salt intake, and chewing khat. It assessed their relationship with significant independent variables. The prevalence of current tobacco use is 2%, which is higher than a previous nationwide survey in Ethiopia (1.4%) (29). However, it is lower compared to studies in Sub-Saharan Africa (2.4%) (5), Nepal (8.9%) (30), and Uganda (15.5%) (31) among women of reproductive age. Socio-cultural influences may contribute to this pattern.

Our study found that as the age of reproductive-age women increased, the odds ratio of current tobacco use decreased, indicating that older women have lower odds of current tobacco use compared to younger women. This finding aligns with a previous study in Nigeria (32). This trend may be attributed to younger populations being more exposed to tobacco advertising and promotion (33), particularly in the African region where the tobacco industry targets vulnerable groups (34). Factors such as weak enforcement of smoke-free zones, ineffective monitoring of policy effectiveness, and limited availability of tobacco cessation treatments at certain sites could also contribute to this pattern (33). In contrast, a previous study in Ethiopia noted that the majority of smokers were in older age groups. This could be due to older women having longer smoking histories, previous unsuccessful quitting attempts, and skepticism about the benefits of quitting tobacco use (35).

Our study found that current tobacco use was approximately three times more prevalent among rural residents compared to urban residents. This aligns with a survey conducted in Afghanistan (36). This disparity may be attributed to a lack of awareness among rural residents, given that the literacy rate in our study was lower among rural residents compared to urban residents. However, this finding was not supported by a study in Indonesia (37), possibly due to socio-demographic and cultural differences between the study populations, including varying values, customs, and taboos.

The study area where our research was conducted resembled a rural setting with a lower socio-economic status compared to Indonesia. Women from the poorest and poorer households were about two times less likely to use tobacco compared to those from the richest households. This could be explained by the fact that a majority (58%) of the population in the area are Protestant religious followers, and as indicated by another study, conservative Protestants tend to accumulate less wealth (38). Conversely, our findings were not supported by another study in Ethiopia (29) and studies conducted in Nepal and Sub-Saharan Africa (5, 39).

Current tobacco use was about three times more likely among women from households with more than four family members compared to those with four or fewer, consistent with a study in South Africa (40). The possible reason might be higher stress among mothers with more children (40). Respondents from urban areas reported higher smoking rates, similar to findings from a study in Bangladesh (41), where participants from Sylhet were over three times more likely to smoke than those from Dhaka (42). This demographic difference might be due to lower education and awareness about NCDs and their consequences in resource-limited countries like Ethiopia. Women who do not use mass media are more likely to use tobacco, as supported by a study from Sub-Saharan Africa (43). This may be due to lower awareness of tobacco’s health risks. Current tobacco use was 24% less likely among women who received health professional advice, likely because such advice increases awareness and helps prevent smoking or encourages quitting.

Our study found that 7% of participants consumed alcohol, which is lower than global (43%), sub-Saharan Africa (23.9%), and Ugandan (10.3%) rates (41, 44). This difference may stem from variations in health behaviors and socio-demographic and cultural characteristics. Women with secondary education were less likely to drink alcohol than illiterate women, consistent with findings from Kassena-Nankana, Ghana. Higher education might lead to greater health awareness. Alcohol consumption was more common in the higher wealth quintile, aligning with trends in sub-Saharan Africa (5). Young women showed higher alcohol use, likely due to urbanization and peer pressure. Women in functional Women’s Development Associations (WDAs) were less likely to drink, possibly due to positive social pressure from non-drinkers in their network. Basketo residents, housewives, government employees, merchants, those with a family history of non-communicable diseases (NCDs), larger family sizes (>4), and those who did not use mass media were more likely to consume alcohol.

The study found that the prevalence of high dietary salt intake in Ethiopia is very high at 90%, up from 61% in previous national surveys (45). This increase may be due to poor awareness and inadequate intervention strategies. High salt intake was inversely related to age, with prevalence rates of 92.6% among women aged 15–24, 89.3% among those aged 25–34, and 88.4% among those aged 35 and older. It was more common in rural areas (92.2%) compared to urban areas (78.7%). Additionally, women who did not use mass media were more likely to consume high dietary salt. This finding aligns with previous national data showing that 83% of rural and 77% of urban residents add extra salt to their diet (45).

The prevalence of khat chewing among the study participants was 4.4%, consistent with other studies in East Africa and the Middle East, where prevalence ranges from 4.1 to 90% % (46). This rate is lower than findings from other Ethiopian studies: 8.4% % (47), 9.89% (48), and 65.9% (49) in southern areas. The lower prevalence in this study may be due to sociocultural variations and differences in the study population. In many parts of Ethiopia, including the study area, khat chewing is less common and socially unacceptable among females due to cultural taboos. Our study found that younger women were less likely to use khat, consistent with other studies in Ethiopia (47, 49). This may be because younger women are more often under family authority, reducing their exposure to khat. Older women, facing stress, depression, loneliness, and social isolation, might use khat as a coping mechanism. Illiteracy and primary education were significantly associated with higher khat use, aligning with previous findings (47). Lower education levels may correlate with a lack of awareness about khat’s harmful effects. Women from wealthier households were more likely to use khat, possibly because they could afford it, a trend supported by other Ethiopian studies (47, 49). Women without health professional advice, those not in the Women Development Army, and those lacking social support had higher risks of khat use, echoing findings from various Ethiopian studies (49). Lack of social support can lead to psychological distress, prompting khat use as a coping strategy. Higher social support and community cohesion likely provide better health information and services access, promoting overall health (51). Women with no mass media exposure were more likely to use khat, possibly because mass media improves health awareness and behaviors. This finding is consistent with a study in the Halaba zone (49). Urban women were about three times more likely to use khat compared to rural women, a finding in line with national studies (52) but not supported by studies in the Halaba and Illu Aba Bor (50) zones. This discrepancy may be due to sociocultural differences and khat availability. Women from smaller households (≤4 members) had higher odds of khat use, possibly due to more available resources. Women with a family history of non-communicable diseases (NCDs) were also more likely to chew khat.

### Clustering of behavioral risk factors

The results show that 90.9% of the population has at least one behavioral risk factor (≥1 RF). Clustering of NCD risk factors decreased with age and wealth among the study population. This aligns with trends in low and middle-income countries but contradicts the Nepal Demographic Health Survey 2016 (30). Younger populations might be more exposed to tobacco and alcohol advertising (33) due to targeted marketing (34) and weak law enforcement, especially in Africa. Socioeconomic variations and Westernized diets in specific areas like the Gofa zone also contribute to higher risk factor clustering, particularly among rural residents. Women without mass media access or knowledge of risk factors, lacking social support, or not part of the women’s development army, were more likely to exhibit NCD risk factors. This highlights the awareness gap and the need for social support. Additionally, women with a family history of NCDs, housewives, merchants, widowed/divorced, and single women showed higher risk. The co-occurrence of risk factors (39) emphasizes the need for integrative interventions to address multiple risk factors. These NCD risk factors can lead to adverse health outcomes, including maternal death (53), posing a significant challenge to achieving Sustainable Development Goals (SDGs) (54).

### Strengths and limitations

This study in Gofa and Basketo zones explores the prevalence and factors linked to NCD prevention among reproductive-aged women. It stands out for its community-based approach, covering rural and urban areas for broader applicability. Rigorous methods were used, including pilot testing, comprehensive training of data collectors and supervisors, and achieving a high response rate (99%), enhancing data quality. Weighted analysis enables extrapolation to the entire study area, examining socioeconomic and knowledge-related factors. However, its cross-sectional design limits the ability to establish causality.

## Conclusion

The prevalence of behavioral NCD risk factors among reproductive-aged women in Gofa and Basketo zones is high, with 91% having one or more risk factors. The most common were high dietary salt intake, alcohol consumption, khat chewing, and tobacco use. Over 10% had two or more risk factors. Tobacco use was more prevalent among rural residents and those without professional advice. Alcohol use was linked to lower education, residence in Basketo, housewives, merchants, government employees, non-members of functional WDA, and those with a family history of NCDs. Younger age, higher household wealth, larger family size, and lack of mass media use were associated with both tobacco and alcohol use. High dietary salt intake was more common among younger women, those with lower education, rural residents, Gofa residents, non-mass media users, those without social support, and those with a family history of NCDs. Khat use was associated with older age, lower education, higher wealth, no mass media use, urban and Basketo residence, smaller family size, lack of professional advice, non-membership in functional WDA, no social support, and family history of NCDs.

Clustering of risk factors was linked to rural residence, younger age, Gofa zone residence, merchants or housewives, lack of social support, non-membership in functional WDA, non-use of mass media, poor knowledge of NCD risk factors, and being widowed/divorced or single. To lessen the expanding NCD epidemic and associated risk factors, national and subnational health authorities should work with the appropriate sectors to effectively monitor the implementation of the policies that have been developed including Food and Medicine Administration Proclamation no. 1112/2019, Media Proclamation no. 1238/2021 and the 2021 Tobacco Control Directive no. 771 issued by EFMHACA. In addition, healthcare professionals need to concentrate on ongoing awareness-raising campaigns that target young people and rural residents who do not have access to mainstream media. Furthermore, the community as a whole need to engage in functional WDA, mass sport campaigns, and NCD awareness-raising programs.

## Data Availability

The raw data supporting the conclusions of this article will be made available by the authors, without undue reservation.
